# Successful sequential chemo-immunotherapy and reduced-volume brachytherapy for bulky residual cervical tumor after external beam radiotherapy: two case reports

**DOI:** 10.3389/fimmu.2025.1687247

**Published:** 2025-11-19

**Authors:** Lan Zhang, Conghui Ai, Shuhui Yu, Kangming Li, Meiping Jiang, Chunfang Zhao, Zheng Li, Xingrao Wu

**Affiliations:** 1 Department of Radiation Oncology, The Third Affiliated Hospital of Kunming Medical University, Yunnan Cancer Hospital, Peking University Cancer Hospital Yunnan, Kunming, China; 2 Department of Radiology, The Third Affiliated Hospital of Kunming Medical University, Yunnan Cancer Hospital, Peking University Cancer Hospital Yunnan, Kunming, China; 3 Department of Gynecologic Oncology, The Third Affiliated Hospital of Kunming Medical University, Yunnan Cancer Hospital, Peking University Cancer Hospital Yunnan, Kunming, China

**Keywords:** locally advanced cervical cancer, bulky residual tumor, chemo-immunotherapy, reduced-volume brachytherapy, case report

## Abstract

**Background:**

Bulky residual tumor after external beam radiotherapy (EBRT) presents a major technical challenge for brachytherapy (BT) and is a significant prognostic risk factor in locally advanced cervical cancer (LACC). The use of chemotherapy combined with an immune checkpoint inhibitor to reduce tumor volume (TV) during the interval between EBRT completion and BT initiation has been rarely explored.

**Case description:**

We present two cases of stage IIIC1r cervical squamous cell carcinoma with residual tumors larger than 5 cm following EBRT. Patient characteristics, examination findings, laboratory and imaging data, treatment responses, and adverse events were recorded. After sequential treatment with albumin-bound paclitaxel plus cisplatin combined with zimberelimab, both patients achieved marked tumor volume reduction and subsequently underwent reduced-volume BT. Neither patient experienced recurrence at 36-month and 25-month follow-ups, respectively.

**Conclusion:**

Sequential chemo-immunotherapy followed by reduced-volume BT may represent an alternative treatment option for patients with bulky residual disease after EBRT. Further clinical studies are warranted to validate the effectiveness of this combined regimen.

## Introduction

Cervical cancer ranks as the fourth most common malignant tumor in women. External beam radiotherapy (EBRT) in combination with concurrent chemotherapy followed by brachytherapy (BT) is the standard treatment for locally-advanced cervical cancer (LACC) (1). In addition to well-recognized prognostic factors, including the International Federation of Gynecology and Obstetrics (FIGO) stage, histology, tumor size, parametrial involvement, lymph node status, and pretreatment hemoglobin levels, tumor regression during EBRT is also an essential outcome predictor (2–6). There is plenty of evidence that tumor volume (TV) parameters at mid-treatment based on magnetic resonance imaging (MRI) have a significant impact on patient survival. Patients with larger high-risk clinical target volumes (CTV_HR_), residual gross tumor volumes (GTVres), or lower tumor volume reduction rates (TVRR) at the first BT session have an increased risk of local failure and distant metastasis (7–9). Although image-guided adaptive brachytherapy (IGABT) and the addition of interstitial needles have improved local control in patients with bulky residual tumors, their application remains limited by technical demands and operator experience. Tumor size reduction according to mid-treatment response can therefore be leveraged to improve prognosis while minimizing the use of interstitial needles and associated complications.

The use of immune checkpoint inhibitor monotherapy represents a major therapeutic breakthrough in LACC. The programmed cell death protein 1 (PD-1) inhibitor pembrolizumab combined with chemoradiotherapy has demonstrated a significant improvement in overall survival (OS), supporting immune-chemoradiotherapy as a standard of care in high-risk LACC patients (10). However, in real-world practice, most patients with LACC do not receive immunotherapy at the beginning of chemoradiotherapy due to financial constraints. The mid-treatment period—when tumor response to EBRT is evaluated—may serve as an appropriate opportunity to determine whether to initiate combined therapy. The combination of chemotherapy and immunotherapy has shown notable antitumor activity in the neoadjuvant setting of LACC (11, 12), but reports describing its use during the interval between EBRT completion and BT initiation remain scarce. In this case report, we describe two patients with LACC who presented with bulky residual tumors after EBRT and were treated with sequential chemo-immunotherapy followed by reduced-volume BT and maintenance immunotherapy. Both patients achieved marked tumor volume reduction after chemo-immunotherapy and attained complete response (CR) during post-BT follow-up. This report aims to provide a clinical reference for managing patients with persistent bulky disease after EBRT.

## Case reports

### Case 1

**Figure 1:**
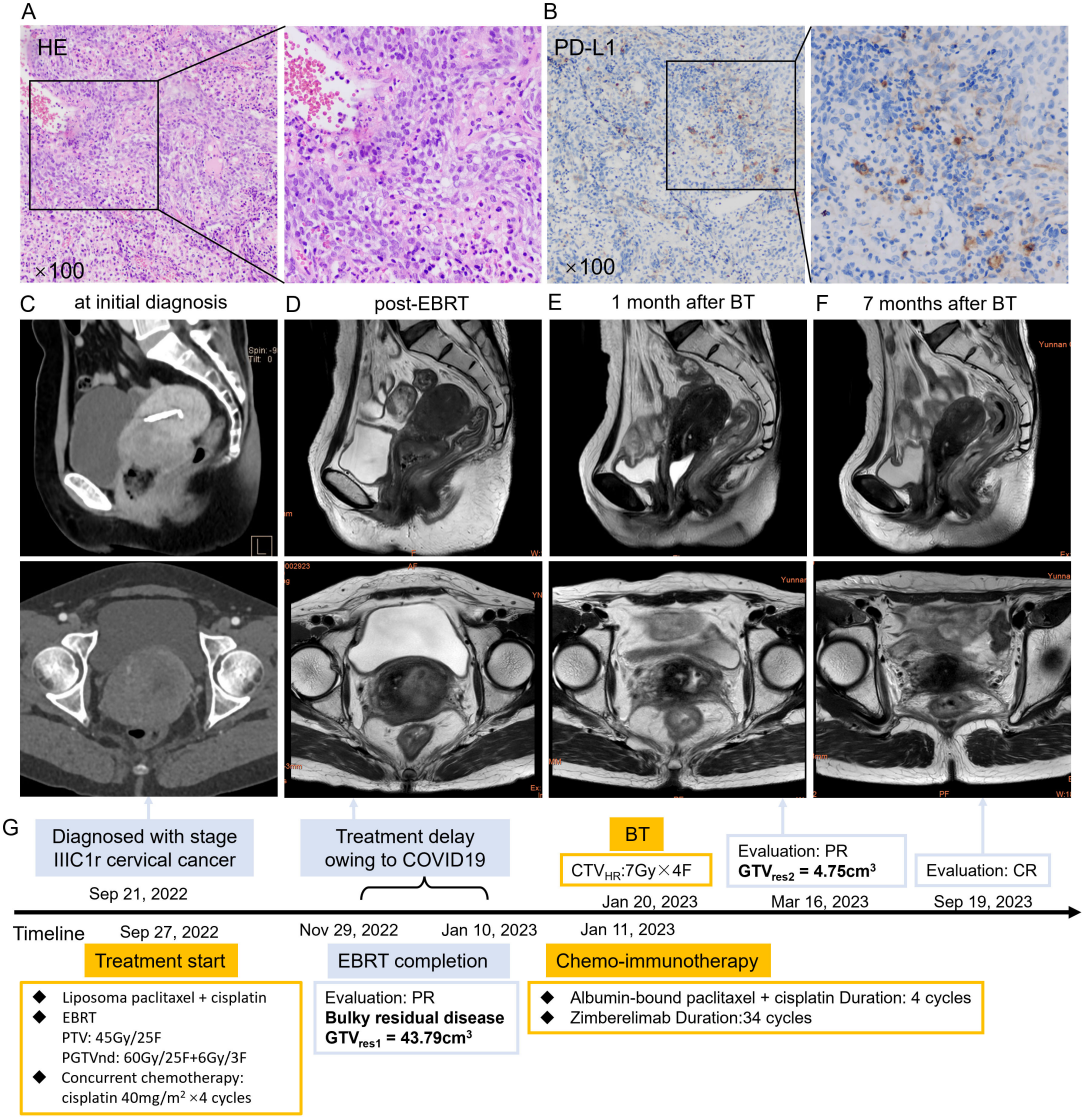
**(A)** HE staining of the patient’s cervical cancer tissue. **(B)** PD-L1 immunohistochemistry staining of the patient’s cervical cancer tissue. **(C)** Sagittal (upper) and cross-sectional view (lower) pelvic CT views at initial diagnosis showing a uterine cervical mass measuring 77×73×39 mm. **(D)** Sagittal (upper) and cross-sectional (lower) pelvic MRI views showing a uterine cervical mass measuring 51×41×40 mm with extensive necrosis post-EBRT. **(E)** Sagittal (upper) and cross-sectional (lower) pelvic MRI views after one month of BT showed marked shrinkage of the cervical mass. **(F)** Sagittal (upper) and cross-sectional (lower) pelvic MRI views after 6 months of BT revealed CR. **(G)** The course timeline illustrating treatment of patient 1. Abbreviations: HE, hematoxylin-eosin; PD-L1, programmed death ligand 1; CT, computed tomography; MRI, magnetic resonance imaging; EBRT, external beam radiotherapy; BT, brachytherapy; CR, complete response; PTV, planning target volume, PGTVnd, planning gross tumor volume-nodal; COVID 19, coronavirus disease 2019; PR, partial response; GTVres, residual gross tumor volume; CTV_HR_, high risk clinical target volume.

A 49-year-old Chinese woman presented to Yunnan Cancer Hospital on September 19, 2022, with a one-month history of abnormal vaginal bleeding and a newly identified cervical mass. The patient (gravida 3, para 3, abortion 0) denied any relevant personal or family history of cancer or other medical or surgical comorbidities. Gynecological examination revealed an exophytic, friable cervical mass (7 cm) involving the vaginal fornix and parametrium. Cervical biopsy confirmed moderately differentiated squamous cell carcinoma (**Figure 1A**), and immunohistochemistry showed programmed death ligand 1 (PD-L1) (Roche/Ventana SP263) combined positive score (CPS) = 10 (**Figure 1B**). Contrast-enhanced computed tomography (CT) revealed marked cervical enlargement measuring approximately 77×73×39mm (**Figure 1C**) and pelvic lymph node enlargement measuring 15×11 mm. Pelvic MRI could not be performed because of a copper intrauterine device (IUD). The patient was diagnosed with stage IIIC1r cervical cancer according to the 2018 FIGO system. Chemotherapy was initiated on September 27, 2022, as follows: 135mg/m^2^ liposomal paclitaxel on day 1 plus 70mg/m^2^ cisplatin on days 1–3. After a 3-week interval, the patient underwent extended-field volumetric modulated arc therapy (VMAT) covering the pelvic and retroperitoneal nodal regions with planning target volume (PTV) dose total (DT) = 45Gy/25F and planning gross tumor volume-nodal (PGTVnd) DT = 60Gy/25F, starting October 20, 2022. A boost dose of 6Gy/3F was administered in a second course to the metastatic lymph node. The treatment regimen included weekly cisplatin (40 mg/m² × 4 weeks). During the first week of EBRT, the patient experienced acute vaginal hemorrhage leading to anemia (hemoglobin 65 g/L). Hemostasis was achieved through therapeutic transcatheter embolization of internal iliac artery branches, and hemoglobin levels normalized following transfusion. The copper IUD was removed on November 24, 2022.

Subsequent contrast-enhanced MRI on November 29, 2022, showed a cervical mass measuring 51 × 41 × 40 mm [CTV_HR_ = 68.76 cm^3^, GTVres1 = 43.79cm^3^, TVRR1 = 61.85%, GTVres = length ×width × height × π/6, TVRR1 = (TV at diagnosis − TV at first restaging)/TV at diagnosis × 100%] with necrosis and gas accumulation involving the vaginal fornix and left parametrium (**Figure 1D**). Multiple pelvic lymph nodes were observed, the largest measuring 1.0×0.6 cm in the left common iliac region.

The patient returned for further treatment on January 9, 2023, more than 5 weeks after EBRT, due to coronavirus disease 2019 (COVID-19) travel restrictions. The residual disease remained bulky (> 5 cm) with extensive necrosis, indicating a hypoxic microenvironment. Because tumor hypoxia is associated with radioresistance, hydrogen peroxide–soaked gauze was inserted vaginally every other day to remove necrotic tissue and enhance radiosensitivity (13, 14). Chemotherapy with albumin-bound paclitaxel (200mg/m^2^) and cisplatin (50 mg/m²) was started on January 11, 2023, to inhibit tumor proliferation. Zimberelimab (360 mg), a China-developed anti-PD-1 monoclonal antibody (15), was added to improve efficacy based on the proven synergistic effects of chemo-immunotherapy combination (16). The first CT-based high-dose-rate (HDR) freehand BT was performed on January 20, 2023, prescribing 7 Gy to CTVHR using Nucletron interstitial trocar needles and the intrauterine tube of the Nucletron Fletcher applicator (**Supplementary Figure 1A**). CTV_HR_ was defined per GEC-ESTRO recommendations (17, 18). After returning for the Chinese Spring Festival, the patient showed notable tumor regression on gynecological examination. She underwent CT-based BT twice a week, DT = 21 Gy/3 F, from February 3–10, 2023 (**Supplementary Figures 1B–D**). Encouraged by the significant efficacy of chemo-immunotherapy, a second cycle was administered during BT on February 8, 2023. MRI on March 16, 2023, demonstrated marked tumor shrinkage to 21 × 18 × 24 mm ×24mm (GTVres2 = 4.75cm^3^), representing an 89.15% reduction [TVRR2 = (TV at first restaging − TV at second restaging)/TV at first restaging × 100%] compared with the post-EBRT measurement (**Figure 1E**). The apparent diffusion coefficient (ADC) value increased from 0.8μm²/ms (post-EBRT) to 0.88μm²/ms. Because of the remaining lesion, two additional cycles of chemo-immunotherapy were given to enhance local control, and Zimberelimab maintenance therapy continued every 3 weeks for 24 months. The cervical tumor gradually regressed and achieved complete response (CR) 7 months after BT (**Figure 1F**). Trends in TV reduction, fluctuations in squamous cell carcinoma antigen (SCC-Ag), and hematologic parameters are illustrated in **Supplementary Figures 2A, B**, and **3**.

In April 2024, the patient reported activity-related lower back pain. CT revealed heterogeneous bone density and mild compression of the L5 vertebral body. She underwent percutaneous vertebroplasty on August 17, 2024, with good outcome: pain resolved and daily activity resumed. Clinical characteristics, treatment regimen, outcomes, and treatment timeline are summarized in **Table 1** and **Figure 1G**. The patient remains well, with no recurrence or notable symptoms, and is satisfied with the treatment response and ongoing follow-up.

**Table 1:** Clinical characteristics, treatment regimens and outcomes.

	Case 1	Case 2
Clinical characteristics		
Age (y)	49	56
Symptoms	Abnormal vaginal bleeding	Postmenopausal vaginal bleeding
Stage	IIIC1r (FIGO 2018)	IIIC1r (FIGO 2018)
Pathology	Squamous cell carcinoma	Squamous cell carcinoma
Differentiation	Moderate	Moderate
PD-L1 CPS	10	90
Primary tumor size (mm)	77×73×39	53×52×66
Treatment regimens		
Treatment components	Chemotherapy + EBRT + chemo-immunotherapy + BT + chemo-immunotherapy + immunotherapy	EBRT + chemo-immunotherapy + BT + immunotherapy
EBRT dose	45Gy/25F, 1.8Gy/F	45Gy/25F, 1.8Gy/F
Concurrent chemotherapy	Cisplatin, 40mg/m^2^/w × 4 times	Cisplatin, 40mg/m^2^/w × 4 times
Chemo-immunotherapy regiments	Albumin-bound paclitaxel (200mg/m^2^) + cisplatin (50mg/m^2^) + Zimberelimab (360mg), q3w	Albumin-bound paclitaxel (200mg/m^2^) + cisplatin (50mg/m^2^) + Zimberelimab (360mg), q3w
Prescribed BT dose	28Gy/4F, 7Gy/F	28Gy/4F, 7Gy/F
Total CTV_HR_ D_90_ in EQD_2_	87.63Gy	86.23Gy
Cycles of chemo-immunotherapy before BT	1	2
Cycles of chemo-immunotherapy during and after BT	3	0
Total cycles of immunotherapy	34	14
Days from EBRT completion to BT initiation	57	47
Days from EBRT initiation to BT completion	113	100
Overall treatment time	27 months	12 months
Outcomes		
TVRR to EBRT, %	61.85	36.89
TVRR to chemo-immunotherapy, %	Not evaluated	88.38
Overall treatment response	Complete response	Complete response
Adverse events of grade 3-4	Compression fracture of L5	Leukopenia, grade 3
Follow up	Progression free for 36 months	Progression free for 25 months

FIGO, International Federation of Gynecology and Obstetrics; PD-L1, programmed death ligand 1; CPS, combined positive score; EBRT, external beam radiotherapy; BT, brachytherapy; CTV_HR_, high risk clinical target volume; D90, dose to 90% of target volume; EQD_2_, equivalent dose in 2Gy fractions; TVRR, tumor volume reduction rate.

### Case 2

**Figure 2:**
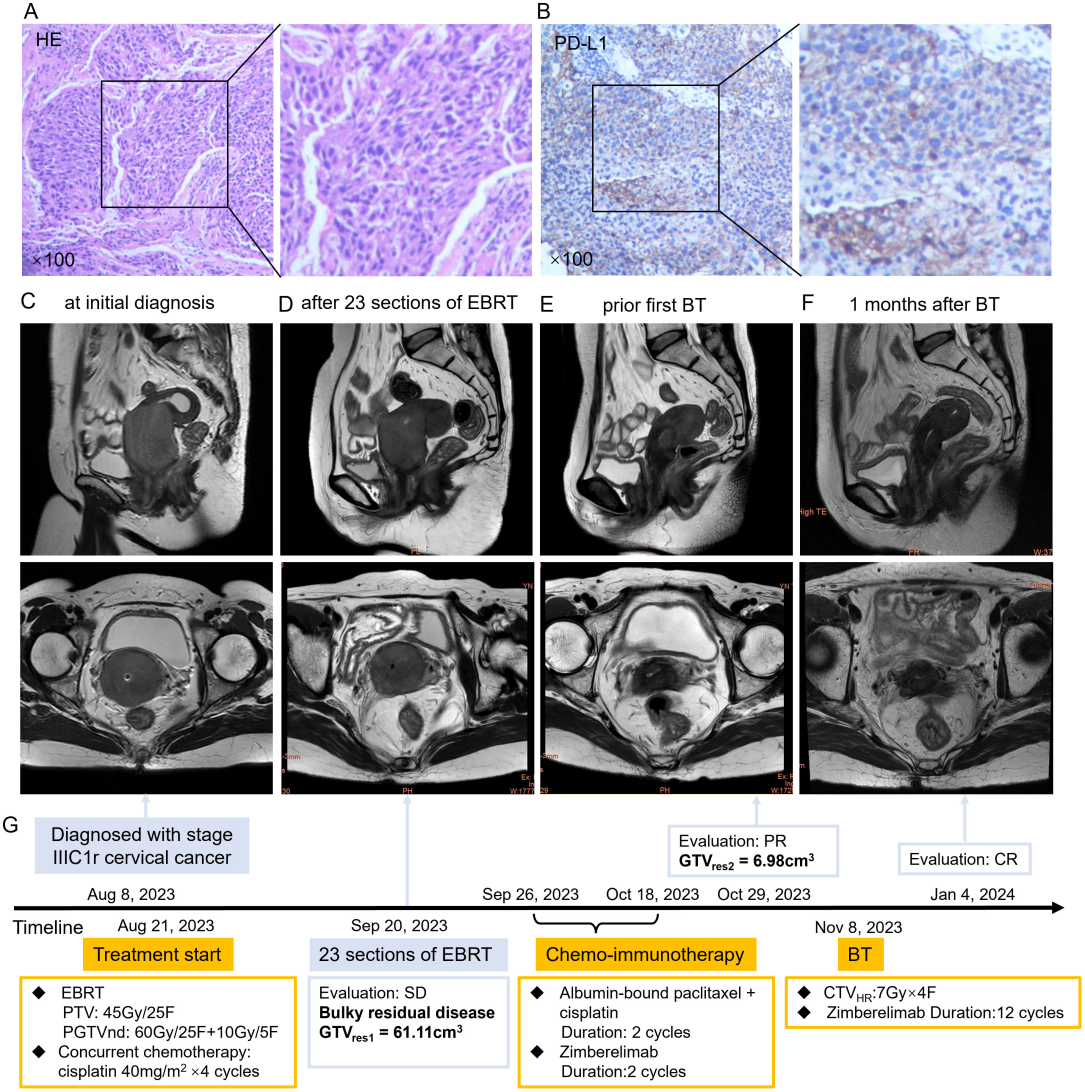
**(A)** HE staining of the patient’s cervical cancer tissue. **(B)** PD-L1 immunohistochemistry staining of the patient’s cervical cancer tissue. **(C)** Sagittal (upper) and cross-sectional view (lower) of pelvic MRI at initial diagnosis showed a mass measuring 53×52×66mm of uterine cervix. **(D)** Sagittal (upper) and cross-sectional view (lower) of pelvic MRI at 23 sections of EBRT showed SD with a mass measuring 50×56×41mm. **(E)** Sagittal (upper) and cross-sectional view (lower) of pelvic MRI after two cycles of chemo-immunotherapy showed marked shrinkage of the cervical mass. **(F)** Sagittal (upper) and cross-sectional view (lower) of pelvic MRI revealed CR after one month of BT. **(G)** The course timeline illustrating treatment of patient 2. Abbreviations: HE, hematoxylin-eosin; PD-L1, programmed death ligand 1; MRI, magnetic resonance imaging; EBRT, external beam radiotherapy; BT, brachytherapy; SD, stable disease; CR, complete response; PTV, planning target volume, PGTVnd, planning gross tumor volume-nodal; GTVres, residual gross tumor volume; CTV_HR_, high risk clinical target volume.

A 56-year-old Chinese woman presented with irregular vaginal bleeding for 4 months, occurring 6 years after menopause. Cervical biopsy performed at a local hospital confirmed moderately differentiated squamous cell carcinoma (**Figure 2A**) with PD-L1 (Roche/Ventana SP263) CPS = 90 (**Figure 2B**). MRI revealed a markedly enlarged cervix measuring approximately 53×52×66 mm, with involvement of the upper one-third of the vagina, lower uterine segment, and bilateral parametria (**Figure 2C**). Multiple pelvic lymph nodes were identified, the largest measuring 16×13 mm. Gynecological examination revealed a firm mass with an ulcerated cavity.

The patient presented to Yunnan Cancer Hospital on August 8, 2023, and was diagnosed with stage IIIC1r cervical cancer per FIGO 2018 criteria. She had three pregnancies (two live births, one abortion) and a history of cholecystectomy 10 years earlier for gallbladder calculi.

Treatment began on August 21, 2023, with DT = 45Gy/25F VMAT covering the uterus, vagina, parametria, and pelvic and retroperitoneal lymph node regions, combined with weekly cisplatin (40 mg/m² × 4). Positive lymph nodes received a simultaneous integrated boost of 60Gy. Pelvic MRI after 23 fractions of EBRT revealed a stable primary disease with 50×56×41mm cervical mass (CTV_HR1_ = 71.86 cm^3^, GTVres1 = 61.11cm^3^, TVRR1 = 36.89%) (**Figure 2D**) and a stable metastatic lymph node (16 × 16 mm).

A large CTV_HR_ at BT is a well-defined risk factor for local failure and distant metastasis (4, 19). In order to reduce TV before BT, sequential treatment was initiated with two cycles of albumin-bound paclitaxel 200mg/m^2^ and cisplatin 50mg/m^2^, followed by Zimberelimab (360 mg) starting on day 4 after EBRT completion. A second-course boost radiotherapy of 10 Gy/5 F was delivered to the involved lymph node from October 24, 2023. Two weeks after completion of the second cycle, pelvic MRI on October 29, 2023, showed a marked reduction in cervical mass (20×23×29 mm, CTV_HR1_ = 10.69 cm^3^, GTVres2 = 6.98cm^3^, TVRR2 = 88.38%) compared with post-EBRT imaging (**Figure 2E**). ADC value increased from 0.6 μm²/ms (post-EBRT) to 0.9 μm²/ms. The metastatic pelvic lymph node also shrank significantly, with the short-axis diameter decreasing from 1.6 cm to 0.7 cm, indicating partial response.

The patient underwent freehand BT (DT = 28Gy/4F once weekly) beginning November 8, 2023 (**Supplementary Figure 4**). Radiographic evaluation 1 month after BT confirmed CR (**Figure 2F**), and no further chemotherapy was administered. Zimberelimab continued during and after BT for 14 cycles in total.

The patient developed grade 3 leukopenia during treatment and moderate anemia (hemoglobin 66g/L) 3 months after BT, likely secondary to prior bone marrow suppression. Hemoglobin normalized after red blood cell transfusion and remained stable thereafter. No residual tumor, local recurrence, or distant metastasis was observed during the 25-month follow-up. Clinical characteristics, treatment regimens, outcomes, disease regression, and hematologic changes are summarized in **Table 1**, **Figure 2G**, and **Supplementary Figures 5** and **6**.

## Discussion

The present case reports appear to be the first describing sequential chemo-immunotherapy and interval reduced-volume BT for bulky residual cervical tumors after external beam radiotherapy (EBRT), in contrast to the current standard of consecutive BT. Although chemo-immunotherapy delayed BT initiation, the residual tumor volume (TV) markedly decreased during this interval, substantially facilitating BT delivery due to the reduced technical complexity of interstitial BT. These findings suggest an alternative treatment option for locally advanced cervical cancer (LACC) that remains bulky after EBRT.

EBRT with 45-50Gy at 1.8-2Gy per fraction, concurrent weekly cisplatin (40 mg/m²), followed by high-dose-rate (HDR) BT to an equivalent dose in 2 Gy fractions (EQD_2_) of at least 80Gy for the residual disease, remains the standard treatment for LACC. Despite receiving the recommended treatment, patients with poorly responsive tumors after EBRT have worse survival outcomes. MRI-based volumetry at 45–50 Gy is widely accepted as a measure of treatment response (20). Jong et al. (21) showed the mid-RT TV>3cm^3^ and TVRR ≤ 87% were independent and strong prognostic parameters in cervical cancer. Kyu et al. (22) reported mid-RT tumor size ≥ 4 cm and TVRR ≤ 90% were significant unfavorable prognostic factors for progression-free survival (PFS). Antoine et al. (23) reported that GTV volume>7.5cm^3^ or reduction <90% at BT significantly reduced overall survival (OS), PFS, local control, and distant metastasis control. Similarly, Minkoff et al. (24) demonstrated thatGTV > 7.5 cm^3^ at the first BT correlated with decreased 2-year local control, PFS, and OS. The EMBRACE-I study also confirmed that CTV_HR_>45cm^3^ was a significant risk factor for local failure (4). TV associated biomarkers identified at mid-treatment as survival predictors in the past 10 years are summarized in **Table 2**. For patients who respond poorly to EBRT, standard treatment remains insufficient, and strategies to enhance treatment efficacy are warranted.

**Table 2:** Tumor volume associated parameters at mid-treatment for survival prediction in cervical cancer.

Study	Year	Country	Study	Stage	Pathology	No. patients	Significant parameters
Johannes Knoth, et al^a^ (19)	2025	Multicenter	Prospective	IB-IVA, defined IVB (FIGO 2009)	Sq, Adeno, AdSq	1318	CTV_HR_>45cm^3^ (HR = 1.93 for 5-year DM)
Yoo Kyung Choi, et al. (5)	2024	South Korea	Retrospective	IB3-IVA (FIGO 2018)	Adeno	102	TVRR≥81.8% (HR = 0.42 for PFS)
Maximilian P. Schmid, et al^a^ (4)	2023	Multicenter	Prospective	IB-IVA, defined IVB (FIGO 2009)	Sq, Adeno, AdSq	1318	CTV_HR_>45cm^3^ (HR = 1.64 for 3-year LC)
Chang Sun, et al (9)	2022	China	Retrospective	IIA-IVA (FIGO 2018)	Sq	217	mid-RT TV>11.38cm^3^ (HR = 3.192 for OS), TVRR≥82.19% (HR = 0.603 for OS; HR = 0.204 for local failure-free survival)
Wenjuan Chen, et al (3)	2022	China	Retrospective	IIA-IVA (FIGO 2009)	Sq	203	Post-EBRT tumor diameter ≤2.4cm (HR = 0.376 for 5-year OS), TVRR<80.1% (HR = 2.998 for 5-year PFS)
Tianyang Ke, et al	2022	China	Retrospective	IB2-IVA (FIGO 2018)	Sq	93	GTV_res,BT1_ volume >8.37cm^3^ (for 2-year LC and OS), GTV_res, BT1_ volume>65cm^3^ (for 2-year PFS) (HR values were not provided)
Jun-Hyeok Kang, et al (6)	2020	Korea	Retrospective	IIB-IVA (FIGO 2014)	Sq, Adeno, AdSq	398	TVRR ≤ 26% (HR = 2.16 for PFS, HR = 2.53 for OS)
Antoine Schernberg, et al (23)	2018	France	Retrospective	IB1-IVB	Sq, Adeno and others	247	TVRR<90% (HR = 7.84 for OS, HR = 4.13 for PFS, HR = 8.45 for LC, HR = 7.03 for DM)
Jong Hoon Lee, et al (21)	2017	Korea	Retrospective	IB2-IVA	Sq	231	TVRR <87% (HR = 3.435 for OS), mid-RT TV>3cm^3^ (HR = 3.106 for PFS)
Kye Chan Lee, et al (22)	2017	Korea	Retrospective	IB1-IVB	/	40	TVRR <90%, mid-RT tumor size>4cm (for PFS) (HR values were not provided)

a results from the EMBRACE-I study.

FIGO, International Federation of Gynecology and Obstetrics; Sq, squamous; AdSq, adenosquamous; CTV_HR_, high-risk clinical target volume; HR, hazard ratio; DM, distant metastasis; LC, local control; TVRR, tumor volume reduction rate; PFS, progression free survival; OS, overall survival; RT, radiotherapy; TV, tumor volume; GTV_res,BT1_, residual gross tumor volume in the first brachytherapy; GTV, gross tumor volume; BT, brachytherapy.

Evidence suggests that dose escalation benefits patients with post-EBRT residual disease by improving local control and specific survival. Combined intracavitary-interstitial (IC/IS) BT can widen the therapeutic window by 5–10 Gy in large tumors and increase local control by approximately 10% (25–27). However, the technical and operational complexity of IC/IS BT limits its routine application. Previous investigators have attempted to simplify the IC/IS technique. Shun et al. (28) reported that combining conventional intracavitary BT (ICBT) with complementary applicator-guided intensity-modulated radiotherapy (IMRT) boosts provided a less invasive and more applicable approach. Recently, customized three-dimensional (3D)-printed applicators have been developed to reduce technical complexity (29–31). It should be noted that BT is technically less challenging for smaller-volume tumors. Currently, there is limited research exploring strategies to reduce tumor size prior to BT.

This study found that chemo-immunotherapy administered after EBRT effectively reduced residual TV before BT, thereby improving local control. Radiation is known to increase PD-L1 expression in multiple cancers, including cervical cancer (32, 33). Immunotherapy following EBRT may therefore enhance the antitumor efficacy of PD-(L)1 inhibitors owing to higher tumoral PD-L1 expression (16, 34). As the regresses macroscopic disease may shrink to microscopic or subclinical disease, requiring a lower radiation dose. Consequently, the dose delivered to adjacent normal tissues is reduced, implying a lower risk of radiation toxicity. Despite a 6–8 week treatment delay, both cases in this study demonstrated excellent local control. We hypothesize that the anti-PD-1 component enhanced radiation-induced immunogenic cell death and that the rapid tumor shrinkage during chemo-immunotherapy mitigated accelerated repopulation.

The advantages of sequential chemo-immunotherapy and reduced-volume BT lie in its efficacy and accessibility. Multiple research efforts have sought to enhance the efficacy of standard concurrent chemoradiotherapy for LACC. The OUTBACK study, which evaluated adjuvant chemotherapy following chemoradiotherapy, failed to demonstrate improved outcomes (35). A key distinction between our approach and the OUTBACK trial was the targeted delivery of adjuvant therapy to high-risk patients with bulky residual disease. In contrast, the INTERLACE study, which used induction chemotherapy before chemoradiotherapy, and the KEYNOTE A-18 study, which incorporated immunotherapy into concurrent chemoradiotherapy, both showed improved survival (10, 36). However, in these studies, patients received induction chemotherapy or immunotherapy regardless of their EBRT response. In our approach, only patients with poor EBRT response were selected for sequential therapy, potentially sparing chemoradiotherapy-sensitive patients from unnecessary additional treatment. Furthermore, Chinese-developed immune checkpoint inhibitors are widely available and comparatively affordable, making this approach feasible for adoption in most healthcare settings.

Patients in this study became anxious when MRI scans revealed bulky residual tumors after EBRT and strongly desired more effective treatment options. Although they understood that combining chemo-immunotherapy might increase the risk of adverse events and prolong treatment, they ultimately chose to proceed. Fortunately, both patients achieved remarkable tumor regression with tolerable toxicity. Neither experienced immune-related adverse events such as rash, thyroid dysfunction, colitis, hepatitis, arthritis, or adrenal insufficiency. The L5 compression fracture in Case 1 may have been caused by osteoporosis that developed after radiotherapy; normal function was restored following vertebroplasty. The anemia observed in Case 2 was likely secondary to post-chemoradiation myelosuppression and resolved after transfusion. At 3-year (Case 1) and 2-year (Case 2) follow-ups, both patients remained disease-free with excellent functional outcomes, maintaining an Eastern Cooperative Oncology Group (ECOG) performance status of 0. They successfully returned to normal social activities and reported a high quality of life. Both patients expressed willingness to share their treatment experiences publicly.

The main limitation of this treatment modality is that the addition of chemo-immunotherapy may result in higher hematologic toxicity. Studies incorporating additional chemotherapy into concurrent chemoradiotherapy regimens have demonstrated increased myelotoxicity (35, 36). Case 1 did not exhibit any myelotoxicity during or after treatment, likely due to the extended interval between EBRT completion and chemo-immunotherapy initiation, as well as the reduced chemotherapy dose. In contrast, Case 2 underwent chemo-immunotherapy 3 days after EBRT completion and developed grade 3 leukopenia despite reduced-dose chemotherapy. An appropriate treatment interval and dose adjustment may help minimize hematologic adverse events.

Another limitation was the absence of MRI evaluation before BT for Case 1. The patient received one cycle of chemo-immunotherapy before BT, and tumor size was estimated by gynecological examination rather than imaging, precluding accurate volumetric assessment of treatment response. In addition, one cycle of chemo-immunotherapy before BT may have been insufficient, as residual disease persisted several months after BT. The optimal number of chemo-immunotherapy cycles and the best intervention timing remain to be determined. Furthermore, the definition of bulky residual disease varies across studies. At our center, this approach is reserved for a small subset of patients with residual tumor diameter >5cm and/or extensive parametrial, bladder, or rectal invasion after EBRT. The > 5 cm threshold, which exceeds the definition of bulky disease in previous studies, was empirically determined based on our clinical experience.

Lastly, although chemo-immunotherapy showed a promising antitumor efficacy, it may not be suitable for all populations. The KEYNOTE**-**826 study showed that patients with PD-L1–negative tumors did not benefit from anti–PD-1 therapy, suggesting that PD-L1 status influences immunotherapy response (16). Besides the biomarker PD-L1 expression, factors such as immune cell abundance and frequent gene mutations (e.g., STK11) have also been associated with chemo-immunotherapy response (12). Laboratory analysis of immune-related biomarkers could therefore help guide therapeutic strategy selection.

## Conclusion

Treatment for LACC patients with bulky residual disease after EBRT remains a therapeutic challenge. Our case reports demonstrate that sequential chemo-immunotherapy followed by reduced-volume BT can achieve favorable clinical outcomes. Further research is needed to determine the optimal patient selection criteria, treatment timing, and appropriate chemotherapy intensity.

## Data Availability

The original contributions presented in the study are included in the article/**Supplementary Material**. Further inquiries can be directed to the corresponding authors.
