# Supplementary material for: Successful sequential chemo-immunotherapy and reduced-volume brachytherapy for bulky residual cervical tumor after external beam radiotherapy: two case reports
Source: Front Immunol. 2025 Nov 19;16:1687247. doi: 10.3389/fimmu.2025.1687247 (PMC12672426; doi:10.3389/fimmu.2025.1687247)

Supplementary Figure 1. The applicator position and dose distribution in the first (A), second (B), third (C) and forth (D) brachytherapy plan for the patient in case 1.

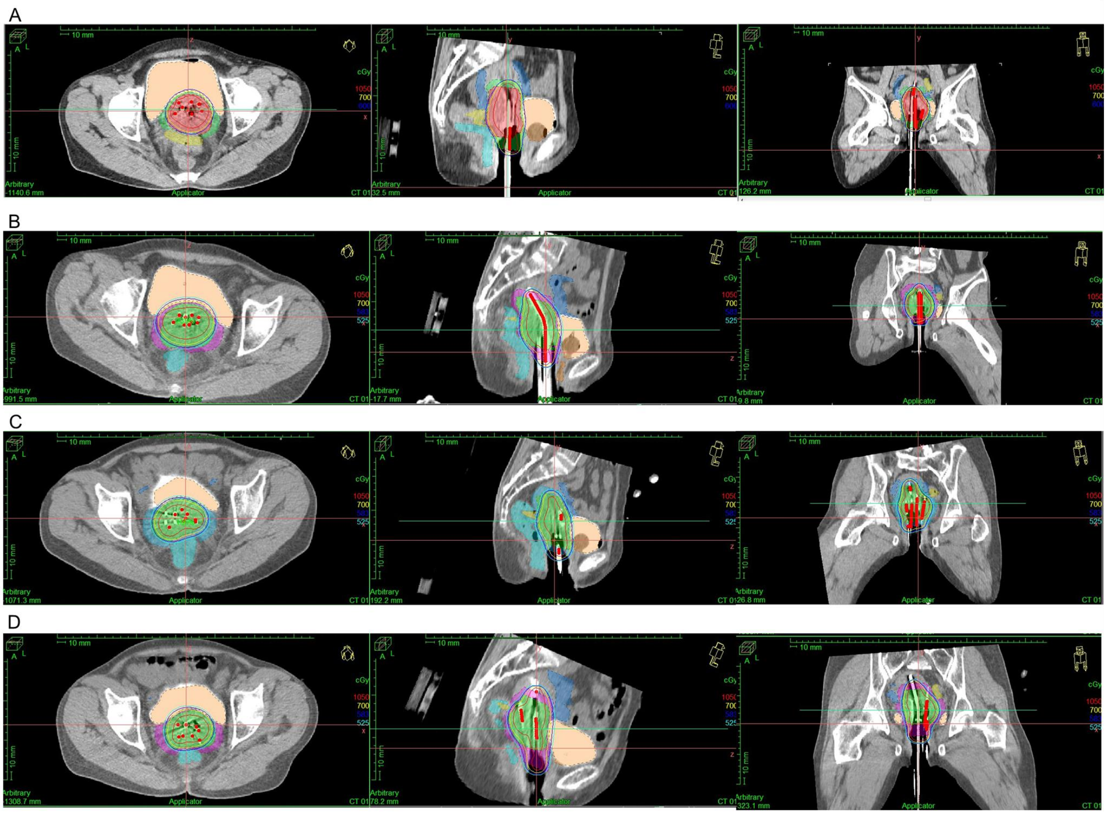

Supplementary Figure 2. **(A)** The trend of tumor volume (TV) reduction and **(B)** fluctuation of squamous cell carcinoma antigen (SCC-Ag) during the patient's treatment.

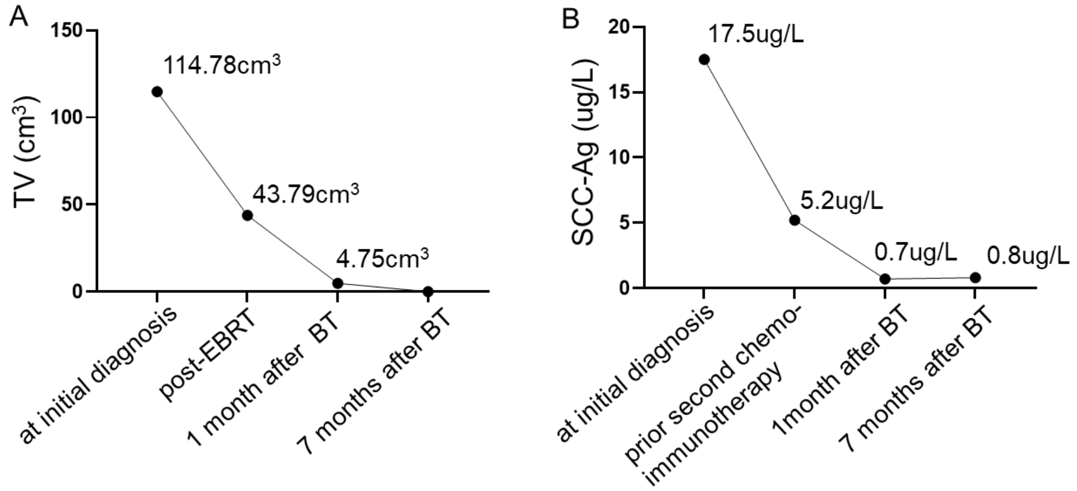

Supplementary Figure 3. The white blood cell (WBC), neutrophil, platelet counts and hemoglobin level of the patient in case 1.

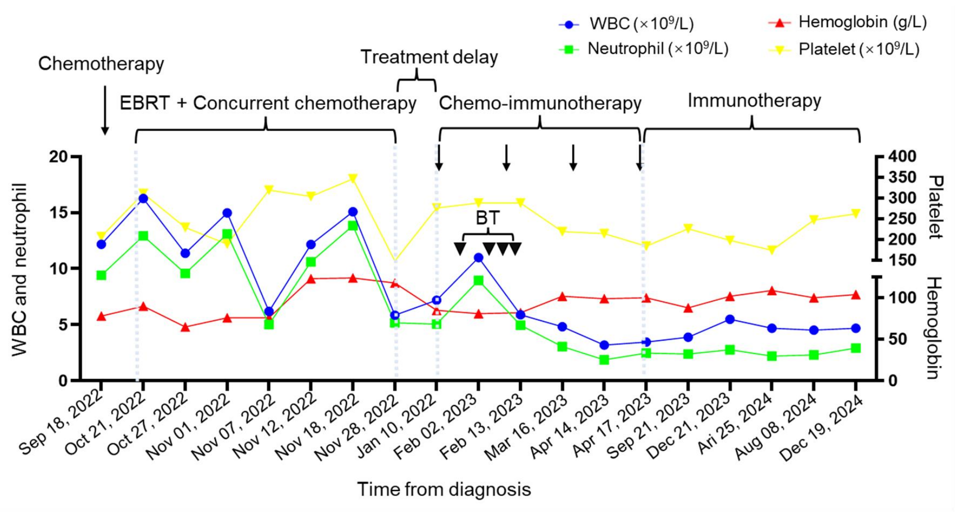

Supplementary Figure 4. The applicator position and dose distribution in the first (A), second (B), third (C) and forth (D) brachytherapy plan for the patient in case 2.

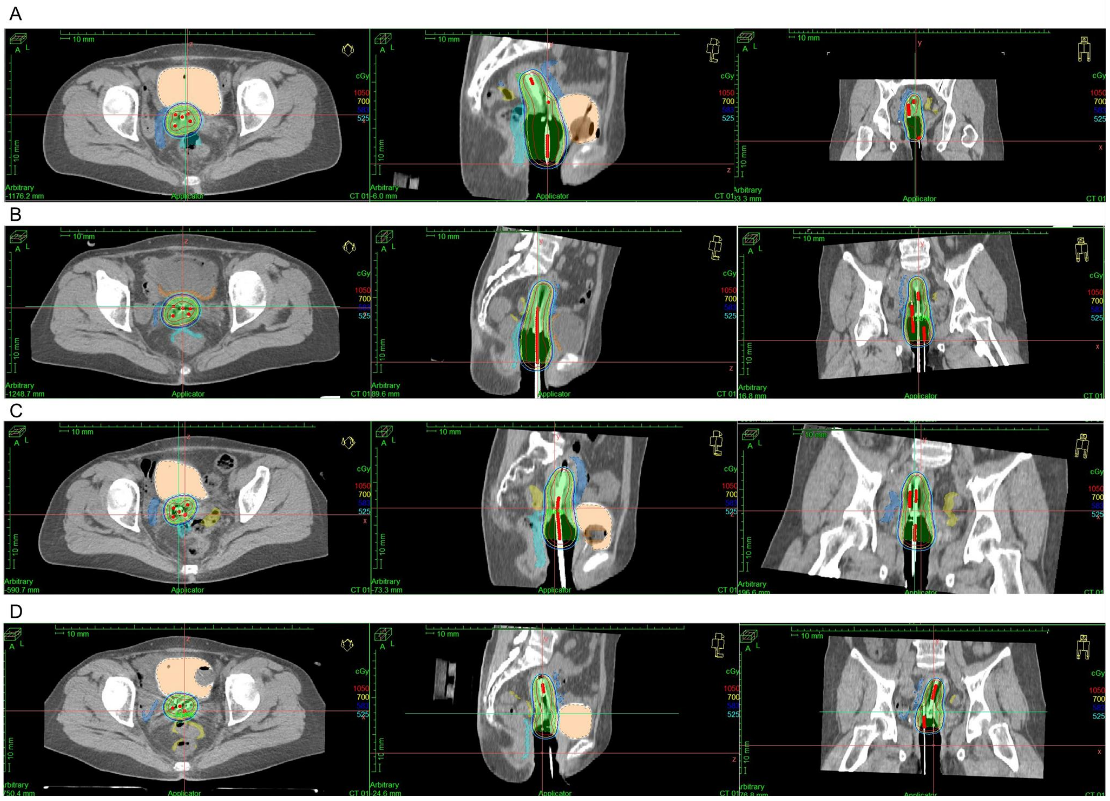

Supplementary Figure 5. **(A)** The trend of tumor volume (TV) reduction and **(B)** fluctuation of squamous cell carcinoma antigen (SCC-Ag) during the patient's

treatment.

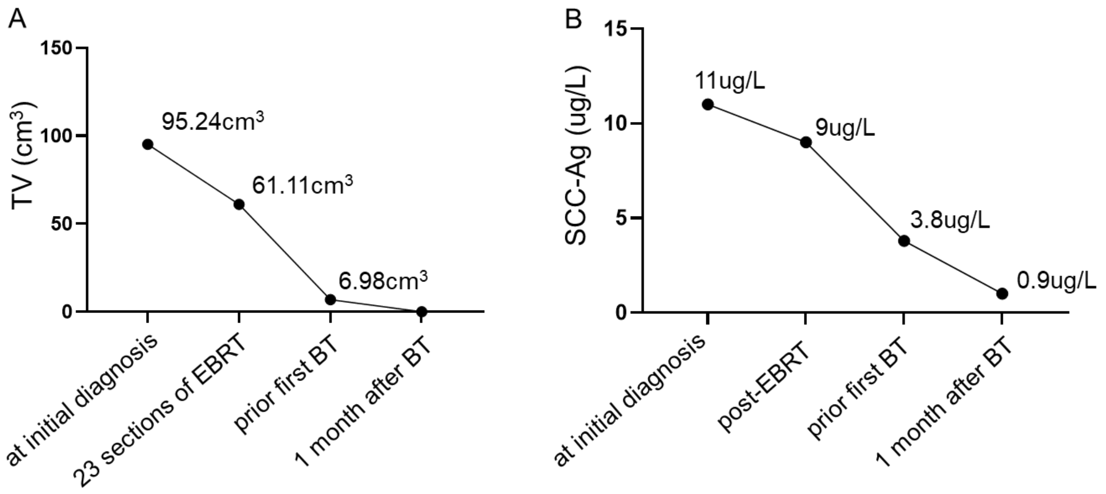

Supplementary Figure 6. The white blood cell (WBC), neutrophil, platelet counts and hemoglobin level of the patient in case 2.

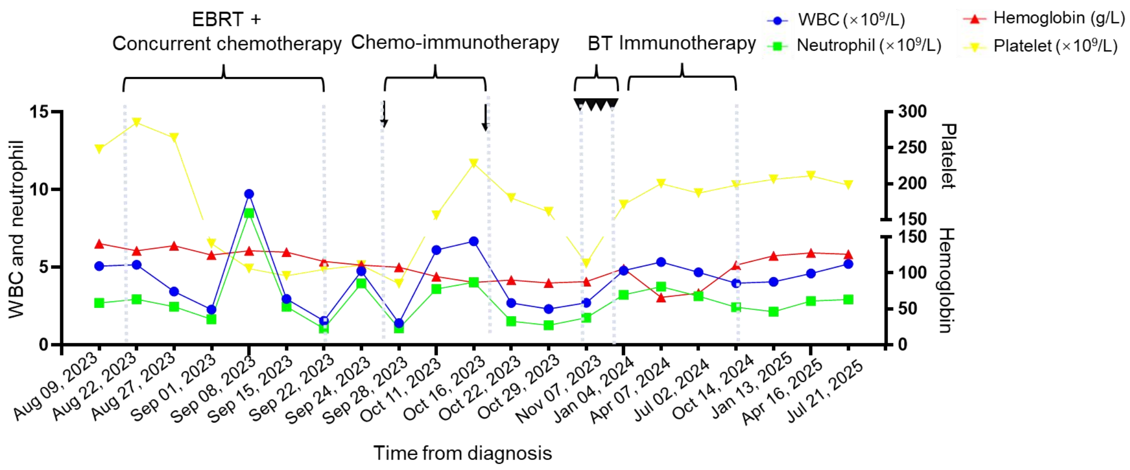

Supplement: Supplementary file 1 [file DataSheet1.pdf]
